# Fabrication and optimization of itraconazole-loaded zein-based nanoparticles in coated capsules as a promising colon-targeting approach pursuing opportunistic fungal infections

**DOI:** 10.1007/s13346-023-01365-0

**Published:** 2023-06-03

**Authors:** Shery Adel, Rania H. Fahmy, Ibrahim Elsayed, Magdy I. Mohamed, Reem R. Ibrahim

**Affiliations:** 1 Department of Pharmaceutics, Faculty of Pharmacy, Ahram Canadian University, 6th of October City, Egypt; 2 Department of Pharmaceutics and Industrial Pharmacy, Faculty of Pharmacy, Cairo University, Kasr El-Aini Street, Cairo, 11562 Egypt; 3 Department of Pharmaceutical Sciences, College of Pharmacy and Thumbay Research Institute for Precision Medicine, Gulf Medical University, Ajman, United Arab Emirates; 4 Department of Pharmaceutics and Industrial Pharmacy, Faculty of Pharmacy, Helwan University, Ain Helwan, Egypt

**Keywords:** Central composite face-centered design (CCFD), Itraconazole, Colon targeting, Colon fungal infections, Zein nanoparticles

## Abstract

Itraconazole (ITZ), a broad-spectrum antifungal drug, was formulated into colon-targeting system aiming to treat opportunistic colonic fungal infections that commonly infect chronic inflammatory bowel diseases (IBD) patients due to immunosuppressive therapy. Antisolvent precipitation technique was employed to formulate ITZ-loaded zein nanoparticles (ITZ-ZNPs) using various zein: drug and aqueous:organic phase ratios. Central composite face-centered design (CCFD) was used for statistical analysis and optimization. The optimized formulation was composed of 5.5:1 zein:drug ratio and 9.5:1 aqueous:organic phase ratio with its observed particle size, polydispersity index, zeta potential, and entrapment efficiency of 208 ± 4.29 nm, 0.35 ± 0.04, 35.7 ± 1.65 mV, and 66.78 ± 3.89%, respectively. ITZ-ZNPs were imaged by TEM that revealed spherical core–shell structure, and DSC proved ITZ transformation from crystalline to amorphous form. FT-IR showed coupling of zein NH group with ITZ carbonyl group without affecting ITZ antifungal activity as confirmed by antifungal activity test that showed enhanced activity of ITZ-ZNPs over the pure drug. Histopathological examination and cytotoxicity tests ensured biosafety and tolerance of ITZ-ZNPs to the colon tissue. The optimized formulation was then loaded into Eudragit S100-coated capsules and both in vitro release and in vivo X-ray imaging confirmed the success of such coated capsules in protecting ITZ from the release in stomach and intestine while targeting ITZ to the colon. The study proved that ITZ-ZNPs is promising and safe nanoparticulate system that can protect ITZ throughout the GIT and targeting its release to the colon with effectual focused local action for the treatment of colon fungal infections.

**Graphical Abstract:**

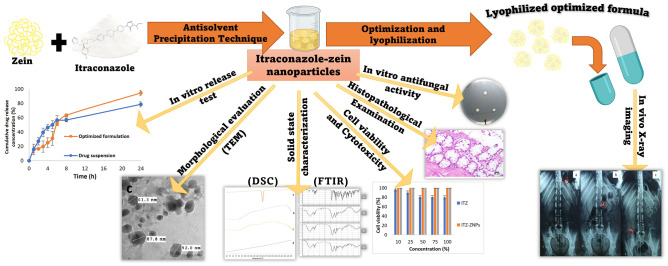

## Introduction

Inflammatory bowel diseases (IBD) patients receiving immunosuppressive drugs usually suffer from opportunistic infections in various areas of the gut, including colonic fungal infections. Such colonic infections usually result in clinical manifestations such as diarrhea, fever, rectal abscess, abdominal pain, and colon ulcers. Accordingly, IBD treatments might frequently incorporate antifungal drugs like itraconazole for patients suffering from opportunistic colonic fungal infections [1–3]. Interestingly, colon cancer patients receiving anticancer drugs (such as quercetin, methotrexate, doxorubicin, and paclitaxel) might also suffer from opportunistic fungal infections due to the immunosuppressive effect of such therapy; therefore, they are at high risk of colon fungal infections. Such fungal infections promote the progress and deterioration of colon cancer; consequently, antifungal treatment might also indirectly aid in amending colon cancer [4, 5].

Poor targeting capabilities of antifungal drugs usually lead to increasing the need for high doses to exert their antifungal activity in the colon; however, this might possibly result in complications and toxicities such as gastrointestinal, kidney, and liver disorders [6, 7]. Consequently, colon targeting and localization of antifungal drugs can significantly reduce the drugs’ doses and so diminish the expected accompanying complications and side effects.

Itraconazole (ITZ), a triazole broad-spectrum antifungal, is used orally for treatment of colonic fungal infections in high doses (200–400 mg/day), which might lead to potential complications such as pseudo-hyperaldosteronism, gastrointestinal, and liver disorders along with induction of congestive heart failure [6–8]. Therefore, reducing ITZ doses achieved by localizing it via colon-targeting therapeutic approach will surely result in reduced complication.

Colon targeting can be classified according to the system unit into single and multiple-unit delivery systems. Various multiple-unit systems, such as liposomes, microspheres, and nanoparticles, have been exploited for colon targeting due to their superior performance over single unit dose systems [9]. *Liposomes* are multi-particulate lipid bilayer vesicles, used successfully for colon targeting and encapsulating either hydrophilic or lipophilic drugs. For example, curcumin-loaded colon-targeting liposomes successfully delivered the encapsulated drug via selective targeting to the colorectal cancer [10]. Also, *microspheres* showed effective colon-targeting capabilities due to their short gastric transition time, fast drug release, especially at the targeting site, resulting in enhanced oral bioavailability of colon-targeted lipophilic drugs [11].

For IBD therapy, it is suggested that *nanoparticulat*e colon-targeting systems are ideal due to their ability to be accumulated exclusively in the inflamed colon tissues [12]. According to their surface modification, they can be classified into pH-dependent, microfold cell-targeted, reactive oxygen species (ROS)-responsive, mucus-permeative, and active targeting-based nano-delivered systems. Such systems are capable distinctively to keep their encapsulated drug from the gastric and intestinal pH and enzymes and releasing it exclusively in the colon [10].

Moreover, colon-targeting delivery systems can be classified according to the targeting technique and the used colon-targeting polymers into time-dependent polymers, microbially triggered polymers, as well as using pH-dependent polymers (such as Eudragit) or a combination of them [13]. Eudragit^®^ (poly-methacrylate) has different grades that include ionic polymers whose solubility is affected by medium pH such as Eudragit® S100, an anionic polymer, that dissolve only at pH > 7 [14]. Unfortunately, although being simple, pH-dependent colon-targeting systems are doubtful due to the highly variable range of physiological and pathological gastrointestinal tract pH. Also, time-dependent colon-targeting systems sometimes show poor colon-targeting capabilities due to the high variability of the transition time throughout the gastrointestinal tract that might lead to imprecise time estimation of the drug release. Recently, a combination of both mechanisms was suggested to ensure an ideal colon-targeting system. For example, indomethacin pellets presented promising colon-targeting system as they targeted indomethacin and sustain its release inside the colon through using Eudragit^®^ FS30D, as a pH-dependent polymer, and Eudragit^®^ RS100, as a time-dependent controlled release polymer [12].

Additionally, the presence of an enormous number of microbiomes and enzymes in the colon promotes using microbially triggered colon-targeting prodrugs. Azo polymer- based hydrogels are microbially triggered prodrugs that succeeded in delivering curcumin effectively to the colonic cancer cells and therefore provided promising colon- targeted cancer treatment [15]. Also, pH–enzyme-sensitive microparticles were fabricated as a specific delivery system for the treatment of ulcerative colitis, based on mesalamine-loaded chitosan microparticles coated with methacrylic acid copolymers. By coating mesalamine-loaded chitosan microparticles with methacrylic acid copolymer, they were able to target mesalamine to the colon efficaciously and promote remedy of ulcerative colitis through using a combination of pH-dependent and microbially triggered colon-targeting polymers [16].

Zein, a natural biodegradable and biocompatible corn protein, is recently used as a colon-targeting polymer for its distinctive ability in protecting drugs during its passage through the gastrointestinal (GI) tract and targeting drug release in the colon [17]. Zein is an amphiphilic protein that contains about 75% hydrophobic amino acids; such hydrophobic regions control the drug release, and additionally, the presence of hydrophilic regions cause its swelling in aqueous media without being eroded making it a suitable excipient for controlled drug delivery systems [18]. All types of zein are insoluble in water but soluble in 60–95% aqueous ethanol solutions. Therefore, it is suggested that zein’s poor water solubility (at pH > 11), in addition to its digestion by enzymes of intestinal fluids, aids in encapsulating lipophilic drugs such as itraconazole, targeting them into the colon, and control their release there [17, 19, 20].

It was expected that using a combination of pH-dependent polymer (Eudragit^®^ S100) in combination with microbially-triggered polymer (zein) can be promising and highly effective for achieving colon-targeting drug delivery [17].

Based on the aforementioned, this study aimed to alleviate colon fungal infections orally through fabrication, characterization, and optimization of colon-targeting coated capsules containing ITZ-loaded zein nanoparticles (ZNPs). Herein, antisolvent precipitation technique was used for preparation of ITZ-loaded ZNPs. Central composite face-centered design (CCFD) was utilized to determine the significant effects of different ratios of zein: drug and aqueous:organic solvents on particle size (PS), polydispersity index (PDI), zeta potential (ZP), and entrapment efficiency (EE %), and to elicit the optimized formulation. Afterward, the optimized formulation was in vitro and *ex vivo* evaluated to ensure its efficacy and safety. Then, lyophilization was done, and the lyophilized formulation was loaded into capsules that were coated with a pH-dependent polymer (Eudragit^®^ S100) to enhance colon targeting. Finally, X-ray examination of the optimized formulation was performed on human volunteers to ensure the ability of the prepared coated capsules to target ITZ to the colon.

## Materials and method

### Materials

Pure itraconazole (ITZ) powder was kindly granted from DBK Pharma Co. (Cairo, Egypt). Zein (protein from corn, pharmaceutical-grade F4400C, approximate molecular weight: 35 KDa) was a kind gift from Flo Chemical Corporation (Qiagen, Germany). Eudragit^®^ S100 was a kind gift from Evonik Industries AG. (Darmstadt, Germany). Sodium lauryl sulfate (SLS) was obtained from oxford lab fine chem (Maharashtra, India). Sodium dihydrogen phosphate, disodium hydrogen phosphate, and absolute ethanol were supplied by El-Nasr Pharmaceutical Chemicals Company (Cairo, Egypt). All other chemicals and solvents were of analytical grade and were used without further purification.

### Application of central composite face-centered design (CCFD)for the optimization of ITZ-ZNP formulation

Design of experiment was employed to determine the statistical significance of individual and combined effects of formulation variables on the experiment outcomes [21]. Subsequently, central composite face-centered design (CCFD) was established to get the least number of experiments needed for optimization and Stat-Ease Design-Expert^®^ software (Version 10.0.0, Stat-Ease Inc., Minneapolis, USA) was used for statistical analysis and optimization [22–24]. In this CCFD, two numerical independent variables were examined: zein:drug ratio (A: 1:1, 5.5:1, 10:1) and aqueous:organic medium ratio (B: 1:1, 5.5:1, 10:1). Table 1 illustrates these independent variables with their generated levels. The monitored dependent variables (responses) were particle size (Y1: PS, nm), polydispersity index (Y2: PDI), zeta potential (Y3: ZP, mV), and entrapment efficiency (Y4: EE, %). According to CCFD, the design matrix is composed of 13 formulations with 5 center points to minimize errors, as outlined in Table 2.

**Table 1:** CCFD independent variables and their generated levels

Factors (independent variables)	Levels of variables		
	**Low (−1)**	**Medium (0)**	**High (+ 1)**
Zein:drug ratio (A, w/w)	1:1	5.5:1	10:1
Aqueous:organic* medium ratio (B, v/v)	1:1	5.5:1	10:1

^*^Organic media refers to 90% v/v ethanol containing 0.5% v/v hydrochloric acid (ethanol-HCl)

**Table 2:** ITZ-ZNP formulations composition generated by CCFD and their resultant monitored dependent variables (responses)

Formula	A: Zein:ITZ ratio (w/w)	B: aqueous:organic* ratio (v/v)	Y1 PS (nm)	Y2 PDI	Y3 ZP (mV)	Y4 EE** (% w/w)
F1	1:1	1:1	169.8 ± 6.03	0.362 ± 0.023	44.2 ± 6.61	31.35 ± 3.25
F2	1:1	5.5:1	199.6 ± 5.79	0.228 ± 0.001	43.8 ± 5.79	60.2 ± 6.84
F3	1:1	10:1	199.2 ± 6.79	0.224 ± 0.008	39.4 ± 3.96	72.80 ± 6.19
F4	5.5:1	1:1	384 ± 2.00	0.508 ± 0.007	46.5 ± 0.57	65.47 ± 3.35
F5	5.5:1	5.5:1	221.38 ± 8.54	0.415 ± 0.009	33.875 ± 5.09	77.74 ± 4.90
F6	5.5:1	5.5:1	208 ± 9.16	0.386 ± 0.009	35.7 ± 1.20	78.39 ± 3.32
F7	5.5:1	5.5:1	268 ± 8.24	0.354 ± 0.003	37.8 ± 2.33	75.93 ± 1.34
F8	5.5:1	5.5:1	235.4 ± 9.29	0.35 ± 0.008	37 ± 0.71	81.54 ± 4.15
F9	5.5:1	5.5:1	227.8 ± 9.87	0.564 ± 0.01	38.4 ± 1.56	79.27 ± 1.34
F10	5.5:1	10:1	181.8 ± 5.69	0.413 ± 0.01	37.9 ± 0.85	64.2 ± 2.47
F11	10:1	1:1	1537 ± 6.17	0.887 ± 0.02	45.6 ± 6.39	80.51 ± 8.52
F12	10:1	5.5:1	288.9 ± 8.95	0.564 ± 0.01	41.6 ± 2.43	66.37 ± 5.29
F13	10:1	10:1	201.8 ± 3.97	0.303 ± 0.006	27 ± 1.72	43.28 ± 4.63

^*^The organic phase refers to 90% v/v ethanol containing 0.5% v/v hydrochloric acid (ethanol-HCl)

^**^PS particle size, *PDI* polydispersity index, *ZP* zeta potential, *EE* entrapment efficiency

Different models, as linear, two-factor interaction model (2FI), and quadratic models, were utilized to achieve the best model for fitting responses, where the best-fitting model for each response was selected according to certain statistical parameters like regression coefficient (*R*^2^) and *P*-value. Also, one way analysis of variance (ANOVA) with 95% confidence interval was conducted for concluding the statistical differences between more than one group and determining each independent variable significance with *P*-values > 0.05 reveals significant difference. Additionally, polynomial regression equations and 3-D surface plots were generated by Stat-Ease Design-Expert^®^ software to determine the effect of the independent variables on responses and figure out the correlations between each response and its significant independent variables.

### Preparation of ITZ-loaded ZNPs

Antisolvent precipitation technique was adopted to formulate ITZ-ZNPs [25]. Briefly, 10 mg of ITZ and the specific amount of zein (according to zein: ITZ ratio presented in Table 2) were accurately weighed and dissolved in certain volume of 90% v/v ethanol containing 0.5% v/v hydrochloric acid (ethanol-HCl) and stirred till dissolved. Drug–zein solution was then added dropwise into accurate volume of distilled water with continuous stirring at 1400 rpm using magnetic stirrer. The volumes of water and organic phase (ethanol-HCl) used differed according to the aqueous: organic phase ratio as presented in Table 2. Afterward, ethanol was slowly evaporated with continuous stirring at 1000 rpm for 2 h at 25 °C. After complete evaporation of ethanol, samples of the prepared formulations were evaluated for their PS, PDI, and ZP. Then, ITZ-ZNP formulations were dried in air and kept in desiccator for further investigations such as EE%. Furthermore, drug-free ZNP formulations were prepared using the same technique for comparison purposes.

### Evaluation of ITZ-loaded ZNPs

#### Particle size, polydispersity index, and zeta potential determination

Particle size (PS), polydispersity index (PDI), and zeta potential (ZP) of each formulated ITZ-loaded ZNPs were measured using photon correlation spectroscopy by Malvern Zetasizer Nano-ZS (Malvern Instruments, Worcestershire, UK). Sample of each formulation was diluted by deionized distilled water and the determination was conducted at 25 °C using a disposable folded capillary cell. The determinations were undergone in triplicates, and the resultant values were expressed as average ± standard deviation (SD).

#### Entrapment efficiency determination

Entrapment efficiency (EE%) was determined using direct method in which the amount of ITZ encapsulated inside the nanoparticles was directly calculated [26]. Briefly, ITZ-loaded ZNPs were centrifuged for 20 min at 13,000 rpm and 4 °C using high speed cooling centrifuge (Hettich Instruments Co., Germany). After centrifugation, the supernatant was withdrawn carefully using sterilized syringe and the separated NPs were left to dry; then, after drying, the dried ITZ-ZNPs were dissolved in specific volume of 90% v/v ethanol and placed for 15 min in an ultrasonic bath sonicator (S30 H, Elma international company, Germany) to ensure complete ITZ solubilization. Furthermore, the same steps were performed for the drug-free ZNPs. So, the amount of encapsulated ITZ was analyzed spectrophotometrically using UV/VIS Spectrophotometer (UV-1800, Shimadzu Corp., Japan) at *λ*_max_ 262 nm. For each formulation, the equivalent ITZ-free zein NP formulation was used as blank. EE% was calculated (as % w/w) using the following equation:

1 $$\mathrm{EE\% }= \frac{\mathbf{A}\mathbf{m}\mathbf{o}\mathbf{u}\mathbf{n}\mathbf{t} \;\mathbf{o}\mathbf{f} \;\mathbf{e}\mathbf{n}\mathbf{c}\mathbf{a}\mathbf{p}\mathbf{s}\mathbf{u}\mathbf{l}\mathbf{a}\mathbf{t}\mathbf{e}\mathbf{d}\;\mathbf{d}\mathbf{r}\mathbf{u}\mathbf{g}}{\mathbf{A}\mathbf{m}\mathbf{o}\mathbf{u}\mathbf{n}\mathbf{t} \;\mathbf{o}\mathbf{f} \;\mathbf{d}\mathbf{r}\mathbf{u}\mathbf{g} \;\mathbf{a}\mathbf{d}\mathbf{d}\mathbf{e}\mathbf{d} \;\mathbf{d}\mathbf{u}\mathbf{r}\mathbf{i}\mathbf{n}\mathbf{g} \;\mathbf{p}\mathbf{r}\mathbf{e}\mathbf{p}\mathbf{a}\mathbf{r}\mathbf{a}\mathbf{t}\mathbf{i}\mathbf{o}\mathbf{n}} \times 100$$

### Statistical optimization and physicochemical characterization of the optimized formulation

Following in vitro evaluation of ITZ-ZNP formulations, Design-Expert^®^ software 10.00 was used for statistical optimization using a set of optimization criteria to conclude the optimized formulation. The optimization criteria were set to the lowest PS, PDI and highest ZP, and EE%; then, desirability values were generated by the software to decide the optimized formulation with the highest desirability factor along with the lowest PS and PDI, and the highest ZP and EE%. The generated dried optimized formulation was then used for further investigations.

#### Differential scanning calorimetry (DSC)

Accurately weighed samples (5 mg) were placed in flat-bottomed aluminum pans that were sealed hermetically. Empty pans were used as reference. Then, pans were heated from 50 to 270 °C with heating rate of 10 °C/min, and a nitrogen purge of 30 ml/min. DSC thermograms of pure ITZ, zein, physical mixture of them, and the optimized ITZ-ZNP formulation were then plotted using differential scanning calorimeter (DSC-60, Shimadzu, Kyoto, Japan).

#### X-ray diffractometry (XRD)

X-ray diffraction pattern was highly preferred in order to understand the particle structure at its atomic level and determine its crystalline state by using X-rays that are characterized by their small wavelengths [27]. Thus, XRD spectra of zein, ITZ, physical mixture of them, and optimized ITZ-ZNPs were analyzed using X-ray diffractometer (X′Pert PRO with Secondary Monochromator, California, USA). The continuous scanning speed of the instrument was set at 0.04°/s with voltage of 45 kV and a current of 35 mA. Cu Kα source was used, and the diffraction peaks detected between 2θ = 2° and 60° with corresponding spacing (d, Å) and relative intensities (I/Io) were obtained.

#### Fourier transform infrared spectroscopy (FT-IR)

FT-IR spectra of pure ITZ, zein, physical mixture of them, and the optimized formulation were examined using FT-IR spectrophotometer (IR Affinity-1; Shimadzu, Kyoto, Japan) to inspect any chemical interaction between ITZ and zein. Potassium bromide (KBr) disc technique was used, and the spectra of samples were performed in the range of 4000 to 500 cm^−1^ with scanning resolution of 2 cm^−1^ [11, 28].

#### In vitro release of ITZ from the optimized ITZ-ZNP formulation

In vitro release of ITZ from the optimized formulation in comparison to ITZ suspension were performed using USP II dissolution apparatus at 50 rpm and a temperature of 37 ± 0.5 °C, where an accurate amount of the optimized formulation, equivalent to 10 mg ITZ, and ITZ suspension (10 mg ITZ suspended in phosphate buffer) were placed in dialysis membrane bag (MWCO = 14 kDa) that was immersed in the dissolution medium using sinker [29–31]. Drug release studies were conducted in 500 ml phosphate buffer pH 7.4 containing 2% SLS to simulate colon pH and achieve sink conditions [7, 32]. Samples of 3 ml were withdrawn from the release medium at (0.5, 1, 2, 3, 4, 5, 6, 8, and 24 h) and replaced with an equal volume of fresh medium and ITZ concentration in the withdrawn samples was determined spectrophotometrically at *λ*_max_ (262 nm). The study was performed in triplicate and the average ITZ released values ± SD at each time interval were calculated.

#### Morphological evaluation using transmission electron microscopy (TEM)

Morphology of the optimized ITZ-ZNP formulation was examined using TEM (JEM 1230 TEM, Tokyo, Japan), where 50 μl of ITZ-ZNPs was placed on a carbon-coated copper grid, left to dry at 25 °C; then, the sample was negatively stained using 2% w/v phosphotungstic acid for about 60 s aiming to reinforce the taken image contrast and boost its quality. Following complete drying, the sample films were visualized, and images were taken by TEM.

### Tolerance studies of the selected optimized formulation

#### *Ex vivo* histopathological examination

A thorough histopathological study was conducted on albino rabbit colon to assess the possible local irritation and ultrastructural changes following ITZ-ZNP administration [13, 33]. Herein, the animal study protocol was authorized by the Faculty of Pharmacy, Ahram Canadian University, Ethics and Research Committee (CEU-522). Briefly, adult male albino rabbit, weighing 2.5–3.0 kg, was euthanized; then, its colon was excised and divided into 3 separated sections after their muscular layers were carefully removed using blunted tweezers. Afterward, each of colon sections was placed in a chamber with 0.63-cm diameter and the colon tissue basolateral side was up and embedded in phosphate buffer (pH = 7.4). These separated colon sections were then soddened with either ITZ-ZNP-optimal formulation, saline (negative control), or DMSO (positive control); the samples were kept on the apical side of the tissue mucosa for 2 h (each sample was done in triplicate). Then, the exposed colon sections were rinsed with phosphate buffer saline (pH = 7.4) and kept in 10% w/v formalin. For histological analysis, each colon section was cut into 4-mm-wide sheets using a microtome and immersed in paraffin wax. Furthermore, these sheets were stained with hematoxylin and eosin to be examined under light microscope to detect any histopathological alterations [34–36].

#### Cell viability and cytotoxicity

Primarily, cell culture of human colorectal cancer (HT-29) cells was procured from Nawah Scientific Inc. (Mokatam, Cairo, Egypt) and was undergone in Roswell Park Memorial Institute (RPMI) media. Herein, HT-29 cells were placed in 96-well plates with RPMI media and seeded with 100 mg/ml of streptomycin, 100 units/ml of penicillin, and 10% of heat-inactivated fetal bovine serum (FBS). Cultured cells were then maintained in humidified, 5% (v/v) CO_2_ atmosphere at 37 °C [37, 38].

Afterwards, the cytotoxicity of the optimized ITZ-ZNP formulation and drug-free ZNPs along with their effects on HT-29 cell proliferation were detected in comparison to pure ITZ using sulforhodamine B (SRB) assay [38]. SRB assay principle is based on the electrostatic and pH-dependent binding between SRB dye and trichloroacetic acid (TCA) fixed cells proteins residues [39]. In brief, samples of cultured cell suspension (about 100 μL ≈ 5 × 10^3^ cells) were incubated in 96-well plates with the above-mentioned media (under cell culture section) for 24 h. After incubation, the cells were further supplied with 100 μL aliquot of media containing different concentrations (10, 25, 50, 75, and 100 μg/ml) of either pure ITZ, optimized ITZ-ZNP formulation, and drug-free ZNPs and kept for 72 h. Then, fixation of cells was performed by adding 150 μL of 10% v/v TCA to replace the medium and incubated at 4 °C for 1 h. The TCA-fixed cells were then rinsed 5 times with distilled water followed by addition of 70 μL aliquot of 0.4% SRB solution and incubation for 10 min in dark place at 25 °C. To enhance the binding between SRB and proteins of TCA-fixed cells, the plates were washed 3 times with 1% v/v acetic acid and dried overnight. Afterward, extraction of SRB from the stained TCA-fixed cells was done by adding 150 μL of 10 mM trichloroacetic acid (TRIS) and the absorbance was measured using a BMG LABTECH – FLUOstar® Omega microplate reader (Ortenberg, Germany) at 540 nm [39]. Ultimately, viability percentages of HT-29 cells upon treatment with pure ITZ, optimized ITZ-ZNPs, and drug-free formulations were determined [37, 39].

#### In vitro antifungal activity

In vitro antifungal activity of the optimized ITZ-ZNP formulation was evaluated in comparison to that of ITZ aqueous suspension. Herein, the sensitivity of *C. albicans* suspension, derived from ATCC® 90,028™ genome, was determined using the disk diffusion method [40]. Briefly, the activated isolate was cultured on Sabouraud’s dextrose agar (SDA) plates, along with 6-mm disks that were previously immersed in either optimized ITZ-ZNP formulation or drug-aqueous suspension. The plates were then placed in the incubator for 48 h at 37 °C. After incubation, inhibition zones around the disks were observed where their diameters were measured and recorded in millimeters. The experiment was done in triplicate and average diameters values along with their standard deviations (SD) were recorded.

### Formulation and evaluation of colon-targeting capsules loaded with optimized ITZ-ZNPs

Primary, lyophilization of the optimized ITZ-ZNP formulation was essential to facilitate its incorporation into hard gelatin capsules. Thus, the optimized ITZ-ZNP formulation was blended with different concentrations of mannitol as cryoprotectant namely 0, 0.25, 0.5, and 1% w/v. Then, samples were frozen in glass vials at −22 °C for 24 h and then lyophilized in Alpha 2–4 LD plus manifold freeze dryer (Alpha 1–4 LSC basic—Martin Christ, Osterode, Germany) with a condenser temperature of −80 °C and under vacuum of 0.1 mbar for 48 h to ensure complete drying. It was important to examine the optimized ITZ-ZNPs PS, PDI, and ZP before and after lyophilization to assess the effect of lyophilization process on formulation parameters. Evaluations were done in triplicate and average values with SD were calculated. Furthermore, the mannitol percentage that maintained the smallest PS, PDI, and highest ZP of formulation was selected for further in vivo examination.

Subsequently, to achieve ITZ colon targeting, capsules (000 size) were loaded with either selected lyophilized optimal ITZ-NPs (equivalent to 10 mg ITZ) or 10 mg of pure ITZ and coated with Eudragit^®^ S100 using coating pan. The used coating solution constituted 10% w/v Eudragit^®^ S100 (as colon-targeting material) and 1% v/v polyethylene glycol 400 (PEG 400, as plasticizer) in 250 ml 90% v/v ethanol. For the success of the coating process, the capsules were loaded into the coating pan so that only one third of the pan was filled, and the coating conditions were adjusted so that the pan rotated at 40 rpm and the coating solution sprayed at a rate of 3.3 ml/min via a peristaltic pump (Minipuls 3, Gilson, France) that was connected to a nozzle of 1-mm pore diameter (Schlick 970, Düsen-Schlick, Germany) and the temperature was kept at 20–25 °C throughout coating. Finally, the capsules were dried using a hot air stream to evaporate the solvent. The coating process was continued until 20-mg increase in weight per capsule was reached [13, 41].

Moreover, colon targeting was ensured through in vitro release test of the coated capsules containing either the optimized ITZ-ZNP formulation (equivalent to 10 mg ITZ) or equivalent amount of pure ITZ. The test was performed in type I dissolution apparatus at 37 ± 0.5 °C and 50 rpm. Where capsules were submerged in 500 ml simulated gastric pH; 0.1 N HCl (pH 1.2) for 2 h, followed by 500 ml simulated intestinal pH (phosphate buffer, pH 6.8) for 3 h, and finally 500 ml simulated colon pH (0.1 M phosphate buffer, pH 7.4) containing 2% w/v SLS for 19 h to indicate the in vivo performance of the colon-targeting capsules [42]. Samples, 3 ml, were withdrawn at predetermined time points of 0.5, 1, 2, 3, 4, 5, 6, 8, and 24 h that replaced by same volume of fresh medium. Afterward, withdrawn samples were filtered and analyzed spectrophotometrically at *λ*_max_ 262 nm. The study was conducted in triplicate and average values with their SD were recorded and the cumulative drug release percentages were calculated and plotted versus time.

#### In vivo X-ray imaging of ZNP-loaded enteric coated capsules

X-ray imaging study was performed to ensure in vivo colon targeting of ITZ-ZNP coated capsules, as well as, to monitor its behavior throughout gastrointestinal tract (GIT). The X-ray imaging protocol was approved by the Faculty of Pharmacy, Ahram Canadian University, Ethics and Research Committee (CEU-522), and the study was performed in line with the principles of the Declaration of Helsinki. Barium sulfate (BaSO_4_) was used as a radio-contrasting agent, aiming to improve the images quality, where the optimized ZNP formulation was formulated using BaSO_4_ instead of ITZ, and loaded into Eudragit^®^ S100-coated capsules using the same preparation technique as previously mentioned [43].

Herein, two healthy human volunteers, aged 30 years old with body weight of 55–60 kg, were well informed of the experiment procedure. Informed consent was obtained from individual participants included in the study. The volunteers were allowed to fast overnight before ingestion of BaSO_4_-ZNP-loaded coated capsules with 200 ml water. Two-hour post-dose, the volunteers had a light breakfast followed by a standard lunch at 4 h post-dose. During the study, radiographs for the whole abdomen were taken at specified time (0.5, 3, and 5 h post-dose) to trace the in vivo behavior and movement of the colon-targeted capsules inside the gastrointestinal tract [44].

## Results and discussion

### Application of central composite face-centered design for the optimization of ITZ-ZNP formulation

Based on the unique ability of zein to self-assemble into nanoparticles that are highly stable in gastric and intestinal fluids while start to lose their integrity upon reaching the colon due to colonic microbiota, thus zein was used as colon targeting microbially triggered polymer [17, 45]. The selection of the most suitable ratios of zein: drug and aqueous:organic phases that were used during formulation is critical as they affect NPs size, stability, drug entrapment efficiency, along with its release from the formulated ZNPs. Thus, to achieve the optimized formulation, with accepted lowest PS and PDI values together with high ZP and EE%, central composite face-centered design (CCFD) was used. The independent variables constituted three ratios of zein:drug (A: 1:1, 5.5:1, 10:1) and three ratios of aqueous:organic media (B: 1:1, 5.5:1, 10:1), and the results of the observed dependent variables (responses) were PS (Y_1_, nm), PDI (Y_2_), ZP (Y_3_, mV), and EE (Y_4_, %) are illustrated in Tables 1 and 2.

### Statistical evaluation of ITZ-loaded ZNPs

#### Particle size (PS), polydispersity index (PDI), and zeta potential (ZP)

The particle size of the formulated ITZ-ZNPs ranged between 169.8 ± 6.03 and 1537 ± 6.17 nm, as shown in Table 2. The 2FI model was suggested with *R*^2^ coefficient of 0.9274. The difference between predicted *R*^2^ (0.7944) and adjusted *R*^2^ (0.9032) was reasonable, indicating the ability of the adopted model to navigate the design space [35, 46, 47]. The generated polynomial equation computes the significance of the independent factors on PS:

2 $$\mathrm{PS }= 332.51 + 243.18\mathrm{A }- 251.33\mathrm{B }- 341.15\mathrm{AB}$$

**Fig. 1:**
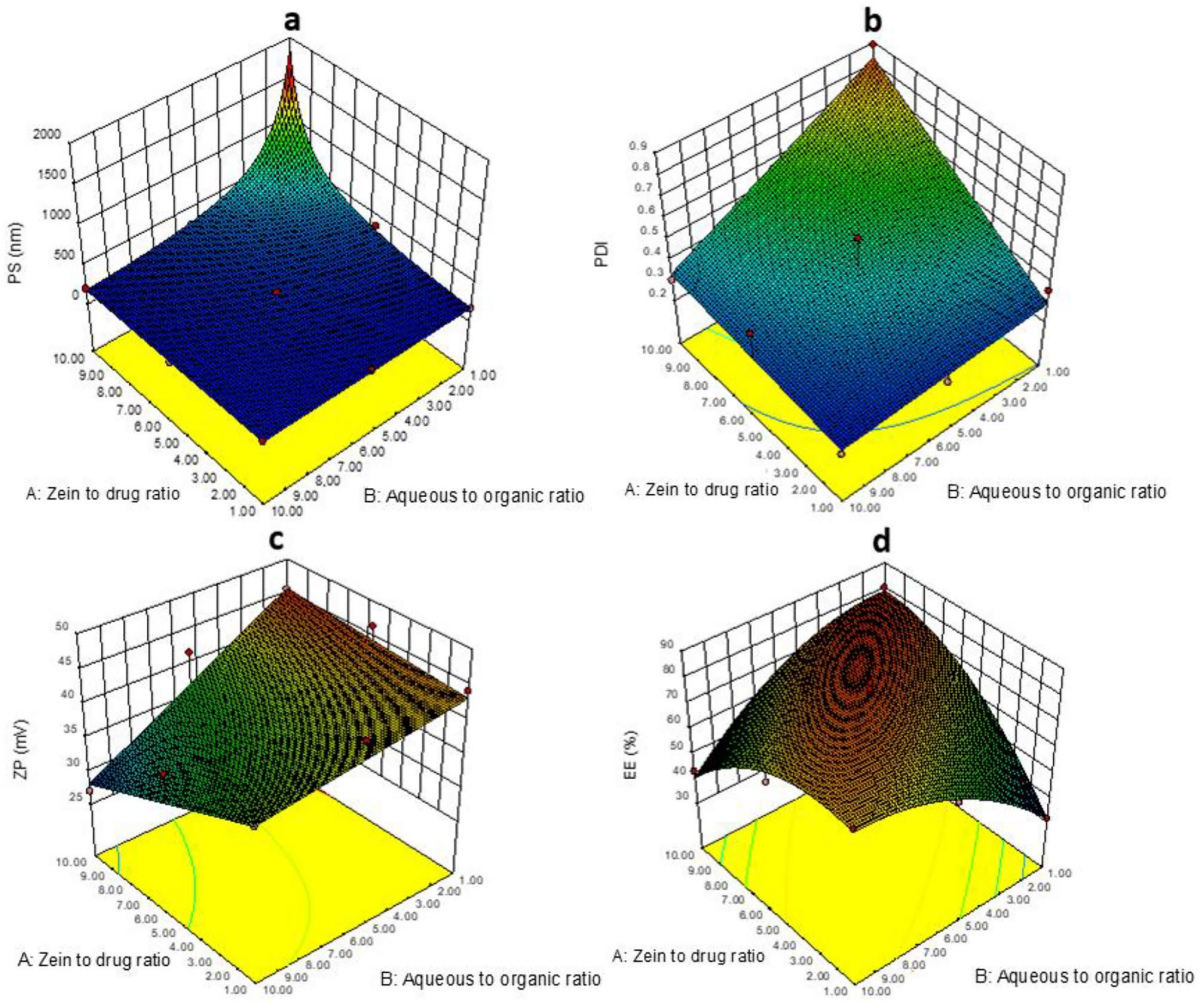
3-D Response surface plots showing the effects of the continuous independent variables; A: zein:drug ratio and B: aqueous:organic ratio on, PS (**a**), PDI (**b**), ZP (**c**), and EE% (**d**)

ANOVA statistical analyses revealed that both variables; zein:drug ratio (A) and aqueous:organic ratio (B) as well as the interaction between them have significant effects on ZNPs PS with *P*-values of 0.0001, 0.0002, and 0.0002, respectively. Figure 1a illustrates that zein: drug ratio (A) has significant positive effect as increasing zein concentration resulted in correlated increase in the particle size. Bisharat et al*.* proved that high zein content might enhance clustering of zein nanoparticles and formation of larger size aggregates [48]. Also, Nunes et al. had similar finding during the formulation of resveratrol-zein nanoparticles [49]. This can be explained by high zein concentration enhancing hydrophobic intermolecular interactions that hampers zein diffusion from ethanol (solvent) into water (antisolvent); thus, when ethanol evaporates, these massive intermolecular interactions led to formation of large aggregates instead of small ZNPs [50].

On the other hand, aqueous:organic phases ratio (B) proved significant negative effect on PS as lowering aqueous phase ratio enhances formation of NPs of large size NP. In accordance with li et al*.* explanation, at low aqueous ratio, precipitation takes place through a nucleation-growth mechanism. In this case, precipitation is an irreversible phenomenon in which an extensive rapid expulsion of water molecules occurs resulting in formation of large size NP. However, high aqueous ratio enhances coacervation of the particles through a nucleation-aggregation mechanism, whereas zein particles grow gradually as ethanol evaporates. Such gradual growth boosts the formation of NP with relatively smaller PS [51]. Thus, increasing aqueous phase ratio had a profound influence in formulating smaller ZNPs.

In addition, the interaction between A and B showed a significant negative effect on PS, where high zein concentration along with high aqueous ratio resulted in small size of ZNP. During self-assembly of ZNP, the presence of huge number of zein molecules in highly hydrophilic aqueous medium resulted in enhancement of hydrophobic interactions between zein molecules that assembled into small spherical NP due to hydrophilicity of the medium [52].

**Polydispersity index (PDI)** is a parameter that demonstrates either uniformity or diversity of NP size distribution. It was ascertained by international standards organizations (ISOs) that PDI values between 0.1 and 0.5 implies monodispersed systems, while PDI values more than  0.7 implies existence of large aggregates that led to polydispersity of the system [53, 54]. Herein, PDI values of the prepared ITZ-ZNPs ranged from 0.224 ± 0.008 to 0.887 ± 0.017 (Table 2). Statistical analysis of the results suggested 2FI model as the best-fitting model for PDI (Y_2_) with model *R*^2^ coefficient of 0.8345. The generated polynomial equation was:

3 $$\mathrm{PDI }= 0.4 + 0.2\mathrm{A }- 0.1\mathrm{B }- 0.1\mathrm{AB}$$

ANOVA statistical analyses presented by the 3-D graphical surface plot (Fig. 1b) proved that incorporation of different ratios of zein: ITZ (A) had significantly positive impact on the PDI (*P*-value = 0.0012). As previously mentioned, there are hydrophobic intermolecular interactions occur between zein molecules, thus increasing zein concentration led to formation of larger zein aggregates that affect dispersity of the system, and consequently, ZNPs of high PDI were obtained.

On the other hand, both aqueous:organic ratio (B) and the interaction between A and B showed significant negative impact on the PDI (*P*-value of B = 0.0029, *P*-value of AB = 0.0242). Similar results by Gajera et al. demonstrated that low aqueous:organic ratio enhanced the agglomeration of particles during nanoparticles formulation leading to increased particle size dispersity throughout the preparation [55]. As well, Danaei et al. found that decreasing aqueous ratio along with increasing zein ratio resulted in formulating NP with large agglomerates and high PDI values [56].

**Zeta potential (ZP)** is an essential criterion when evaluating the physical stability of nanoparticles; it is important to quantify NP surface charges and reveal the physical stability of pharmaceutical nano-systems. As, high ZP values (about ± 30 mV or higher) indicate the presence of enormous charges on the NPs surface that generate electric repulsion force, thus prevent aggregation of NP and increase system stability; however, values around ± 20 mV are still considered as deflocculated stable systems [57–60]. Herein, zeta potential study of the ITZ-ZNPs showed positive values ranged from 27 ± 1.72 to 46.5 ± 0.57 mV, as illustrated in Table 2 indicating that the prepared ITZ-ZNPs are of very good stability as all the prepared formulations were positively charged with around 85% of them had ZP > 35 mV.

The 2FI model was the best-fitting model for ZP (Y_3_) with *R*^2^ of 0.7174. The difference between the predicted *R*^2^ (0.537) and the adjusted *R*^2^ (0.6232) was reasonable, as it is less than 0.2 and implies good ability of model to predict accurate responses values with the ZP polynomial equation:

4 $$\mathrm{ZP }= 39.1 - 2.2\mathrm{A }- 5.3\mathrm{B }- 3.5\mathrm{AB}$$

The polynomial equation together with ANOVA statistical analyses presented by the 3-D surface plot (Fig. 1c) clarified that the aqueous:organic media ratio (B) showed a significant negative effect on the ZP with a *P-*values equals to 0.0033 while zein:drug ratio (A) and interaction between the two independent factors (AB) has no significant effect on the ZP values as its *P*-value was 0.1359 and 0.0654, respectively.

The significant effect of (B) could be explained based on the fact that zein protein acquires positive charges at low pH media (pH 2) [61, 62]. So that increasing the ratio of the aqueous medium to the acidic organic phase (HCl-ethanolic solution) results in diluting the acidity and increasing the media pH causing a corresponding reduction in ZNP-positive charges. While at low aqueous to acidic organic phase ratio, the nanoparticles dispersion pH is more acidic leading to increased positive charges on the ZNPs and accordingly higher ZP.

#### Entrapment efficiency (EE%)

The efficiency of zein protein to entrap ITZ and self-assemble to form ITZ-ZNPs is displayed in Table 2. ITZ entrapment in ZNPs ranged between 31.35 ± 3.25 and 81.54 ± 4.15% w/w. The response surface quadratic model was used to best fit EE% (Y_4_) with regression coefficient *R*^2^ of 0.9773. The predicted *R*^2^ was 0.8678 and the adjusted *R*^2^ was 0.961, implying good model prediction for the response. The EE% polynomial equation was:

5 $$\mathrm{EE \% }= 77.6 + 4.3\mathrm{A }+ 0.5\mathrm{B }- 19.7\mathrm{AB }- 11.7\mathrm{A}2 - 10.2\mathrm{B}2$$

The above equation together with ANOVA statistical analyses revealed that zein:drug ratio (A) had significant positive effect on the EE% with a *P*-value = 0.01 while aqueous:organic ratio (B) has insignificant effect (*P*-value = 0.702). From Eq. (5) and Fig. 1d, it is obvious that increasing zein concentration led to significant increase in the EE% which might be due to the increased hydrophobic interactions between ITZ and zein upon increasing zein concentration, leading to higher percentage of ITZ encapsulated effectively inside ZNPs [63]. Similar findings were observed by Cai et al*.* in formulating pectin-zein nanoparticles for encapsulating the hydrophobic curcumin, where increasing zein content enhanced the interaction between the hydrophobic drug molecule and zein protein resulting in more drug molecules to be encapsulated into the hydrophobic core of ZNPs [62].

### Statistical optimization and physicochemical characterization of the optimized formulation

Stat-Ease Design-Expert^®^ software (Version 10.0.0, Stat-Ease Inc., Minneapolis, USA) was used for optimization of formulation parameters and election of the optimized ITZ-ZNP formulation based on the predetermined criteria (minimum PS and PDI, maximum ZP, and EE%). Following optimization, the optimum formulation with the highest desirability value was selected. As presented in Table 3, formulation with 5.5:1 (zein:drug ratio) and 9.5:1 (aqueous:organic ratio) was considered the optimum formulation with 0.755 desirability as it had the most acceptable combination of responses (expected PS, PDI, ZP, and EE were found to be 196.19 nm, 0.309, 34.47 mV, and 70.22%, respectively).

**Table 3:** ANOVA results of the responses including predicted and adjusted* R*^2^, composition of the optimized formulation and its desirability, expected values, observed values, and residual errors of the responses

Factors	Optimized level (desirability = 0.755)
A: Zein:drug ratio	5.5:1
B: Aqueous:organic ratio	9.5:1

	ANOVA results		
Responses*	Regression coefficient (*R*^2)^	Predicted *R*^2^	Adjusted *R*^2^
Y1: PS (nm)	0.93	0.79	0.90
Y2: PDI	0.84	0.59	0.78
Y3: ZP (mV)	0.72	0.54	0.62
Y4: EE (%)	0.98	0.87	0.96

Optimized formulation		
Expected values	Observed values**	Residual error***
196.19	208 ± 4.29	−11.81
0.309	0.35 ± 0.04	−0.041
34.47	35.7 ± 1.65	−1.23
70.22	66.78 ± 3.89	3.44

^*^PS particle size, *PDI* polydispersity index, *ZP* zeta potential, *EE* entrapment efficiency

^**^Observed value = mean ± standard deviation (*n* = 3)

^***^Residual error is the difference between expected and observed values

Following formulation of the optimized formulation, its observed response values were PS of 208 ± 4.29 nm, PDI of 0.35 ± 0.04, ZP of 35.7 ± 1.65 mV, and EE of 66.7817 ± 3.89%. A comparison between the predicted and observed response values was performed and the residual for each response was calculated to enhance validity of the performed statistical optimizations. The residual errors were found to be 11.81 nm, 0.041, 1.23 mV, and 3.44% for PS, PDI, ZP, and EE, respectively. Such small residual error values indicate that the observed results were very close to the predicted ones, thus ensuring good model fitting and efficient optimization [64, 65].

#### Differential scanning calorimetry (DSC)

**Fig. 2:**
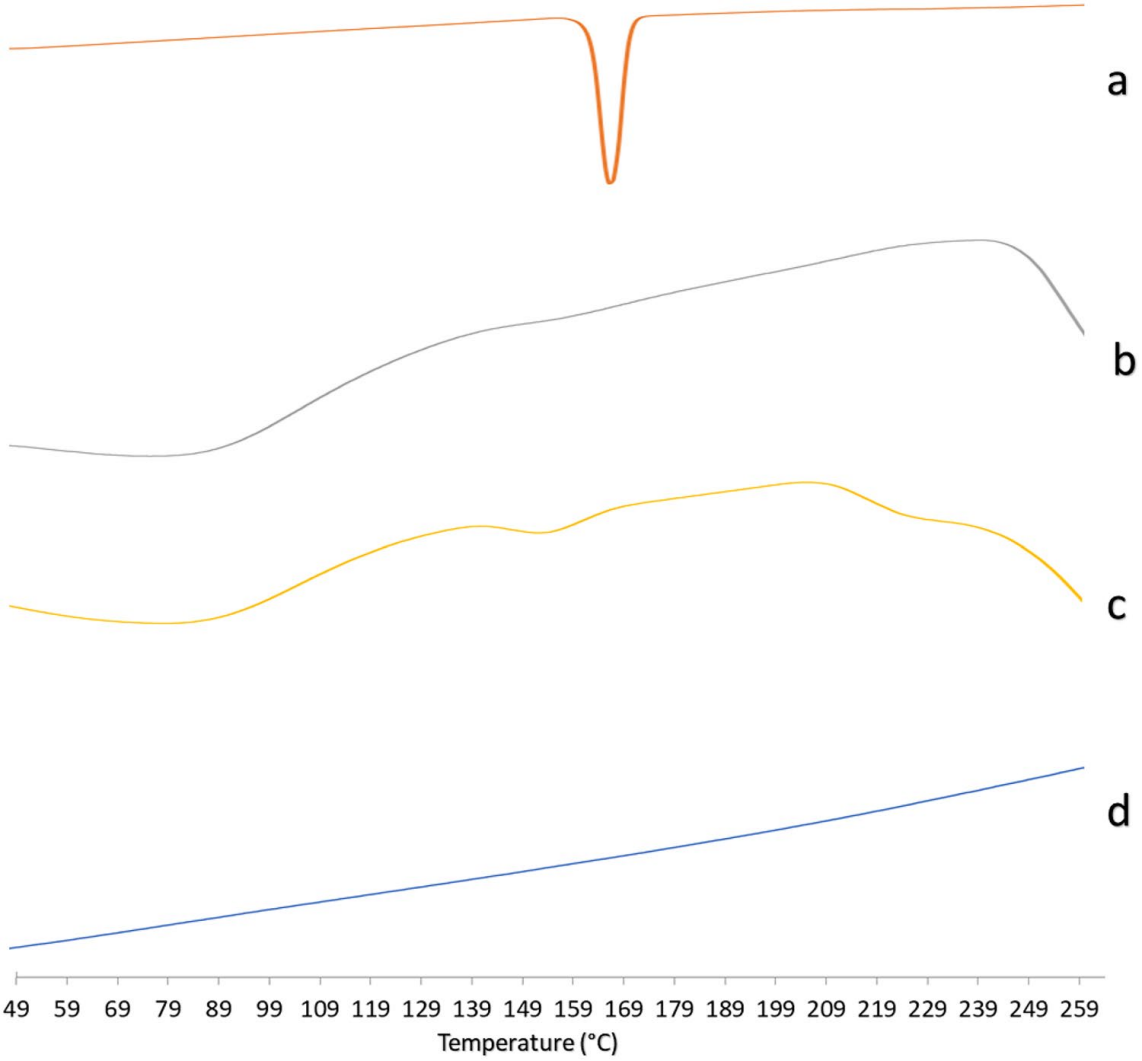
DSC thermograms of pure ITZ (a), zein (b), ITZ-zein physical mixture (c), and ITZ-ZNP-optimized formula (d). The curves have been displaced vertically for better visualization

DSC studies were carried out to show the physical state of ITZ-ZNP constituents and their thermotropic properties, besides, to ensure their degree of purity and check crystallinity [66]. To understand the crystalline nature of ITZ before and after formulation, DSC study for ITZ, zein, their physical mixture, and ITZ-ZNP-optimized formulation was performed, and their thermograms were illustrated in Fig. 2. ITZ thermogram (Fig. 2a) showed a single sharp endothermic peak at 167 °C, such a sharp peak corresponding to its melting that indicates the typical crystalline nature of pure ITZ. Similar endothermic peak for ITZ was observed with Alves-Silva et al*.* who observed that ITZ melting point represented by sharp endothermic peak at 167.9 °C due to ITZ crystallinity [67].

Figure 2b showed the DSC thermogram of zein showing broad endothermic peak at around 80 °C might be due to zein protein degradation from heating during the study. Wang et al. had similar observations as zein powder sample showed broad endothermic peak ranging around 82.09 °C indicating protein degradation without phase transition, as well, such broad peak, means a longer melting process, suggesting a non-crystalline state [68].

In ITZ-zein physical mixture DSC thermogram (Fig. 2c), zein broad endothermic peak was clearly prominent while ITZ peak was present with decreased intensity such decrease in the intensity of the characteristic endothermic peak may be due to the dilution effect due to relatively high zein:drug ratio in the optimized formulation (5.5:1 zein:drug ratio).

On the other hand, Fig. 2d showed complete disappearance of ITZ peak in the optimized formulation, proving ITZ transformation from crystalline to amorphous form which was then molecularly dispersed throughout the ZNP matrix [69, 70].

#### X-ray diffractometry (XRD)

**Fig. 3:**
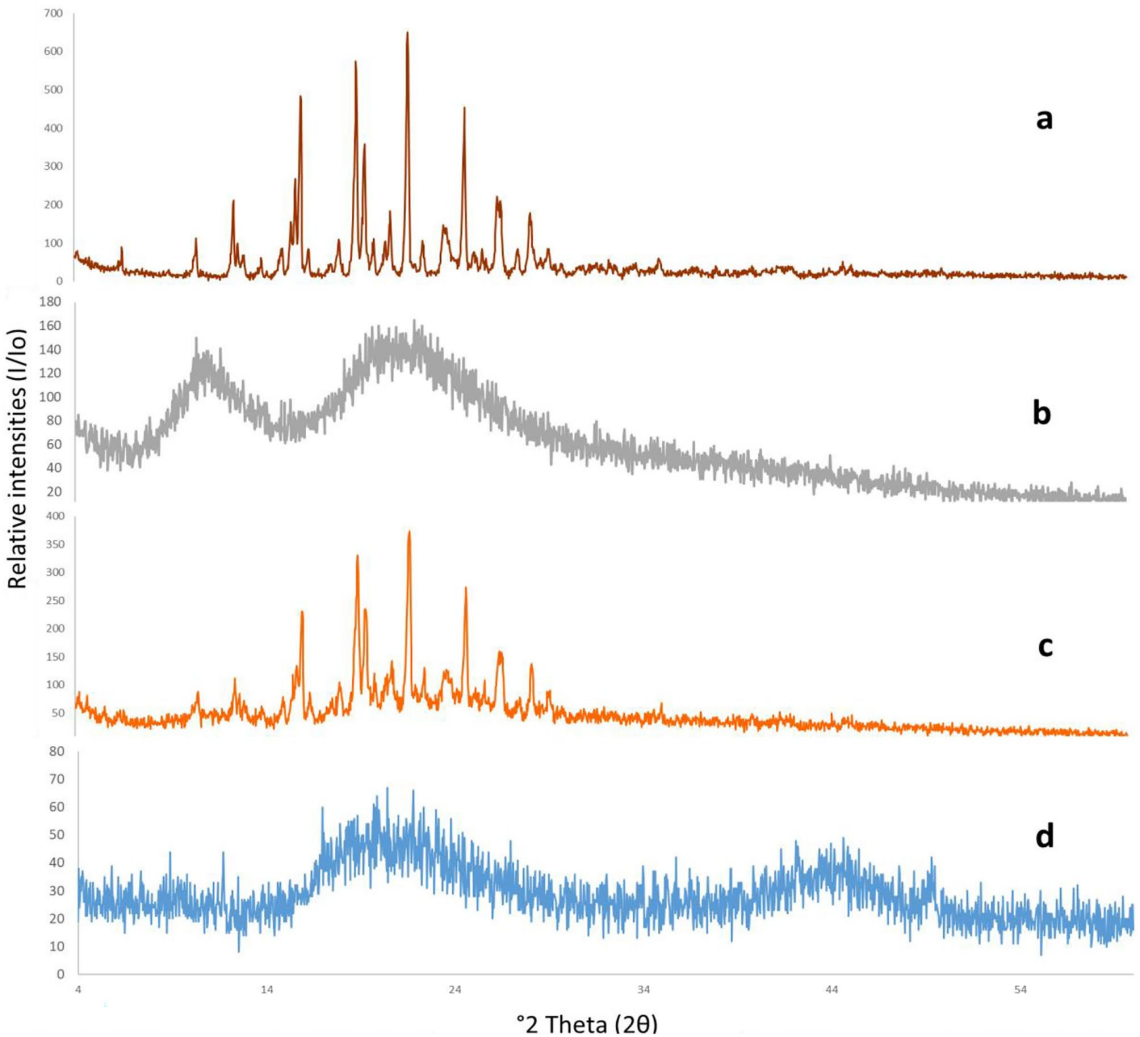
XRD diffractograms of pure ITZ (a), zein (b), ITZ-zein physical mixture (c), and ITZ-ZNP optimized formulation (d). The curves have been displaced vertically for better visualization

XRD analysis was performed in order to examine the inner nanocrystalline structure of ITZ during nanoparticle formulation. Potential changes of ITZ crystalline structure may occur according to its chemical nature and physical hardness inside ZNPs [71]. X-ray diffraction is a well-established tool to study crystal lattice arrangements and it yields particularly useful information on the degree of sample crystallinity that might affect various characteristics such as solubility. The X-ray diffractograms of ITZ, zein, physical mixture of them, and optimized ITZ-ZNPs are presented in Fig. 3. ITZ diffractogram (Fig. 3a) showed several sharp high intensity peaks at 8.75, 10.75, 14.51, 17.54, 20.38, 23.51, 25.42, and 25.11 (2θ) that indicates the presence of well-crystallized pure ITZ of sharp defined diffraction peaks [72]. Figure 3b showed two broad scattering bands, instead of sharp peaks, due to α-helices backbone of the amorphous zein [73]. The physical mixture diffractogram (Fig. 3c) maintained the diffraction patterns of both ITZ and zein, but it was observed that ITZ characteristic diffraction peaks were of lower intensities probably due to the dilution effect (zein:ITZ ratio in the physical mixture was the same as that of the optimized formulation (5.5:1)) [27]. The permanence of these characteristic peaks confirmed ITZ crystalline state and excluded the existence of possible drug–zein interaction in the physical mixture.

On the other hand, the X-ray diffractogram of the optimized formulation (Fig. 3d) showed disappearance of ITZ diffraction peaks. Typically, such diffusive pattern is commonly observed following the conversion of the crystalline ITZ into its amorphous states and its dispersion within zein matrix in amorphous form.

The XRD studies confirmed the DSC results and assured the complete loss of ITZ crystallinity and suggested its inclusion into the ZNP matrix.

#### Fourier transform infrared spectroscopy (FT-IR)

**Fig. 4:**
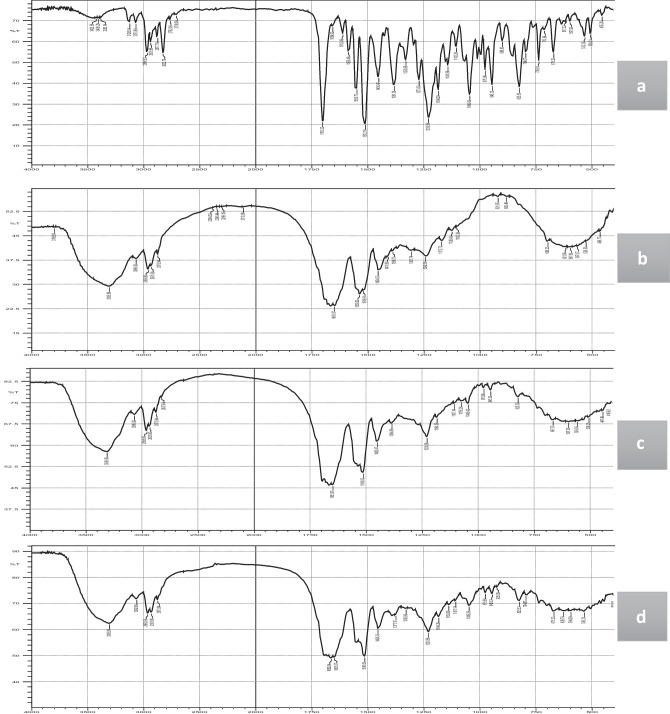
FT-IR of pure ITZ (**a**), zein (**b**), ITZ-zein physical mixture (**c**), and ITZ-ZNP optimized formula (**d**)

FT-IR spectroscopy was employed to elucidate the main structural properties of pure ITZ and zein and then detect any possible chemical interaction by pointing the characteristic modifications of their functional group occurred due to chemical interactions between them upon nanoparticle formulation [74, 75]. FT-IR spectra of ITZ, zein, ITZ-zein physical mixture, and ITZ-ZNP-optimized formulation are displayed in Fig. 4. Figure 4a demonstrates ITZ characteristic peaks at 3000–3100 cm^−1^ (aromatic C-H), 1701 cm^−1^ (carbonyl group), 1550 cm^−1^ (C = N), 1450 cm^−1^ (C = C), and 794 cm^−1^ (C–Cl) [76]. While Fig. 4b shows that zein characteristic peaks are 3305.99 cm^−1^, 2931.8 cm^−1^ (-COOH), 1651 cm^−1^ (C = O stretching), 1539 cm^−1^ (N–H bending), and 1242 cm^−1^ (C-N stretching) [77–79].

It was observed that the spectra of both physical mixture (9.5:1 zein:drug ratio; same ratio as the optimized formulation) (Fig. 4c) and ITZ-ZNP-optimized formulation (Fig. 4d) showed very close IR behaviors to that of zein spectra (Fig. 4b) due to dilution effect on ITZ due to very high zein:drug ratio (9.5:1) in both samples, consequently, ITZ peaks are less observed in both physical mixture and the optimized formulation.

Furthermore, Fig. 4c and d show the characteristic ITZ peaks at 1701 cm^−1^ (carbonyl group) and 1550 cm^−1^ (C = N) disappeared, suggesting coupling of NH of zein with the carbonyl group of itraconazole via hydrogen bond [80, 81]. However, this coupling does not affect the drug antifungal activity as triazole moiety of itraconazole is the functional group responsible for its antifungal activity [82]. To further prove that this interaction did not affect antifungal activity of ITZ, a specific antifungal activity study was performed.

#### In vitro release of ITZ from the optimized ITZ-ZNP formulation

**Fig. 5:**
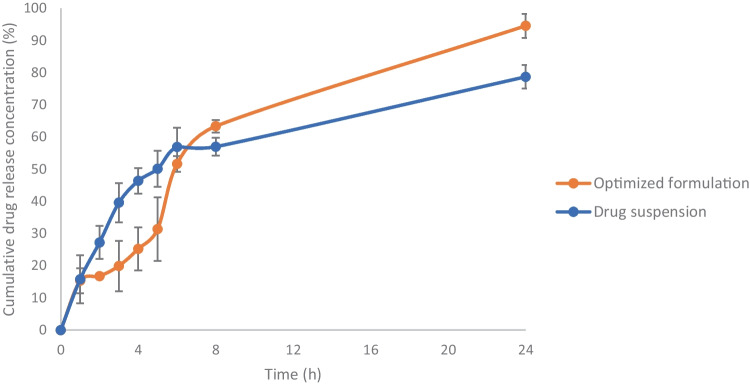
release profile of the optimized ITZ-ZNP formulation compared to pure ITZ suspension in phosphate buffer (pH = 7.4) at 37 ± 0.5 °C. Each point represents mean ± SD (*n* = 3)

In order to evaluate ITZ release from the formulated ZNPs in colon, in vitro release study of the optimized formulation, in comparison to that of pure ITZ suspension, was undergone in phosphate buffer (pH = 7.4) containing 2% SLS at 37 ± 0.5 °C and their release profiles are elucidated in Fig. 5. It was found that within the first 1 h, ITZ release was 15.8 ± 7.5% and 15.3 ± 3.9% from drug suspension and optimized formulation, respectively. These results attributed to initial burst release of adsorbed ITZ on the surface of the optimized ITZ-ZNPs [83]. After 5 h, 50.127 ± 5.6% ITZ was released from drug suspension while only 31.4 ± 9.8% ITZ was released from optimized formulation indicating the ability of ZNPs to initially protect the encapsulated ITZ; however, with time, ZNPs lose its integrity due to formation of aqueous channels leading to swelling of ZNPs facilitating gradual drug release through such pores [84, 85]. After 24 h, 94.5 ± 3.7% ITZ was released from optimized ITZ-ZNP formulation while only 78.7 ± 3.7% ITZ was released from drug suspension. Thus, in vitro release results ensured suitable ITZ release upon reaching the colon. Karthikeyan et al. had similar findings during their study of encapsulating a hydrophobic drug (aceclofenac) in zein microspheres where a gradual drug release occurred due to the presence of hydrophobic interactions between the hydrophobic drug aceclofenac and zein protein [86]. Also, zein hydrophobic matrix prevents its erosion but swell in a hydrophilic like pattern that allow gradual drug release [18].

#### Morphological evaluation using transmission electron microscopy (TEM)

**Fig. 6:**
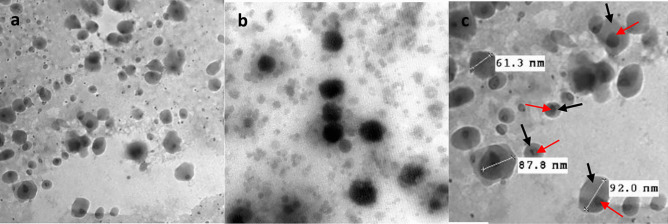
Transmission electron micrographs of **a** ITZ-ZNPs with magnification power of 50,000 × . **b** ITZ-free ZNPs with a magnification power of 80,000 × . **c** Size of ITZ-ZNPs with magnification power 80,000 × , core (red arrows) shell (black arrows)

The morphology of the optimized ITZ-ZNP formulation and ITZ-free ZNPs were observed via TEM and presented in Fig. 6a and b, respectively. The photomicrographs showed that ITZ-ZNPs and ITZ-free ZNPs all have solid dense structures, with homogenous spherical shape, smooth surfaces, and uniform sizes. Moreover, the core–shell structure of ITZ-loaded ZNPs was elucidated in Fig. 6a, indicating the presence of itraconazole in the inner hydrophobic core of zein nanoparticle [87].

It was interesting to notice that PS range measured by TEM was slightly lower than that measured by zeta sizer, as shown in Fig. 6c. This might be due to hydration of the sample before zeta sizer measurement. Upon hydration of the sample prior to zeta sizer measurements, aqueous channels in the hydrated ZNPs were formed by time, resulting in swelling of ZNPs and increasing its PS during measurement. While for TEM, dried ITZ-ZNPs were observed without any hydration; thus, no swelling occurs to the ZNPs [84, 85].

### Tolerance studies of the selected optimized formulation

#### *Ex vivo* histopathological examination

**Fig. 7:**
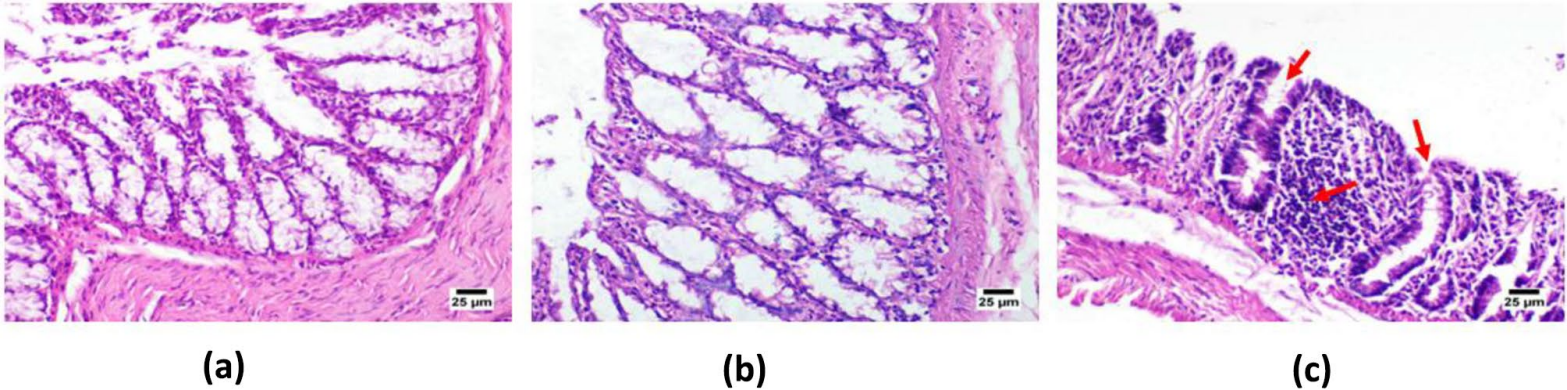
Photomicrograph of rabbit colon mucosa after treatment with optimized ITZ-ZNP formulation (**a**), saline (negative control) (**b**), and DMSO (positive control) (**c**)

Microscopic histological examination of rabbit colon mucosa after treatment with either ITZ-ZNP optimal formulation, saline (negative control), or DMSO (positive control) was performed and presented in Fig. 7 to ensure the tolerance and safety of the formulated optimized ITZ-ZNPs and determine any possible destructive or toxic effects of various ingredients used in their formulation on the rabbit colon tissues.

Microscopic examination of rabbit colon revealed that application of ITZ-ZNPs as well as saline (negative control group), presented in Fig. 7a and b, respectively, showed normal histological structure of the colon wall, normal mucosa with intact goblet cells. Normally, colon wall mucosa is differentiated into lamina muscularis mucosa, glands, and connective tissues. In depth, this structure is constituted of tunica mucosa that was formed of simple columnar epithelium and lamina propria that contained glands with basally situated nuclei and numerous goblet cells [88]. Such structure was totally maintained following administration of either saline as negative control or ITZ-ZNPs indicating that the optimized ITZ-ZNP formulation has harmless innocuous effect on the colon tissue and thus confirmed the safety of their administration.

On the contrary, the DMSO (positive control group, Fig. 7c) exhibited marked histopathological deterioration of the normal histological structure of the colon wall. Necrosis of the colon mucosa was detected in numerous sections associated with inflammatory cells infiltration with accumulation of eosinophilic tissue debris. In conclusion, the optimal ITZ-ZNP formulation was not accompanied by any marked inflammation or necrosis emphasizing its tolerance and safety on colon cells.

#### Cell viability and cytotoxicity

**Fig. 8:**
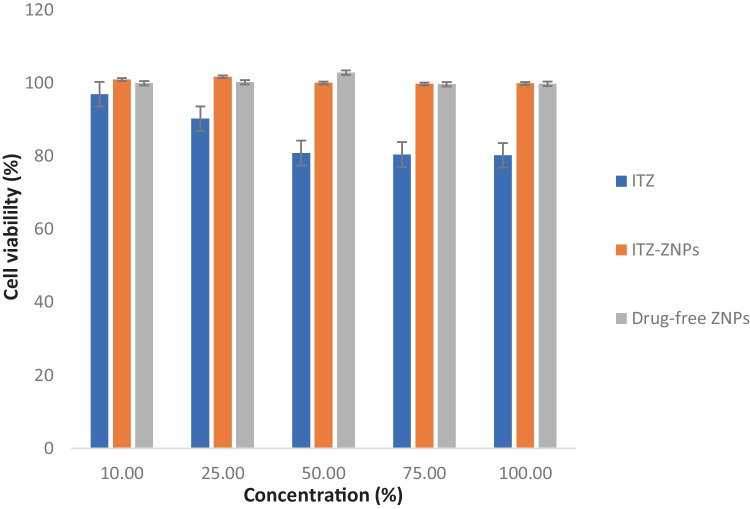
Effects of ITZ, ITZ-ZNPs and drug-free ZNPs on the viability of HT-29 cells at 72 h after exposure, *P* < 0.05. Each point represents mean ± SD (*n* = 3)

SRB assay was preformed to assess biosafety and cytotoxicity of pure ITZ, optimized ITZ-ZNP formulation, and drug-free ZNPs, at different concentrations, against HT-29 colon cancer cells. Figure 8 demonstrates HT-29 colon cancer cell viability after treatment with either ITZ, optimized ITZ-ZNP formulation, and ITZ-free ZNPs for 72 h. The results proved that both ITZ-ZNPs and ITZ-free ZNPs had no cytotoxic effect on HT-29 colon cancer cells at any concentration time point indicating safety of zein as a matrix for encapsulating ITZ. Similar results were proved by Nunes et al., where zein had a low cytotoxic profile, making it a safe matrix for drug delivery systems [49]. However, pure ITZ processed a concentration-dependent cytotoxicity on HT-29 colon cancer cells at 72 h post-treatment. This may be due to ability of ITZ to suppress tumor growth and inhibit cell proliferation by inducing Hedgehog signaling pathway that mediates autophagy cell death of colon cancer cells [38].

Thus, this study proved that ITZ-ZNP is a promising safe system; as ZNPs proved to be a safe nanoparticulate matrix, besides, encapsulating ITZ in ZNPs reduced ITZ cytotoxic effects.

### In vitro antifungal activity

The antifungal activity of the optimized ITZ-ZNPs in comparison to that of pure ITZ aqueous suspension were assessed by disc diffusion method and determination of the inhibition zones around discs in mm. For the optimized ITZ-ZNP formulation, the average value of observed inhibition zones was 15 ± 0.15 mm while that of ITZ suspension was 9 ± 0.1 mm. ANOVA Statistical analysis proved that the inhibition zone observed with ITZ-ZNPs was significantly higher than that of ITZ suspension (*p* < 0.05) which proves that inclusion of ITZ inside the optimized ITZ-ZNPs enhanced the antifungal activity of ITZ.

It was suggested that this enhanced antifungal activity could be due to the enhanced solubility of ITZ by its encapsulation inside zein nanoparticles. As found by Wu et al. that found that encapsulation of essential oils inside zein nanoparticles enhanced their solubility [89]. Also, formulation of ITZ into nanoparticles may enhance its cellular uptake and ITZ accumulation in the fungal cells, consequently its activity was enhanced. Sufia et al. found that entrapment of drug into nanoparticles induces its cellular uptake and tissue entrapment [90].

Consequently, it was concluded that encapsulating ITZ in ZNPs is a promising safe system, as ZNPs were not only safe matrix but also had the ability to reduce ITZ cytotoxic effect along with enhancing its antifungal activity.

### Formulation and evaluation of colon-targeting capsules loaded with optimized ITZ-ZNPs

In order to select the optimal concentration of mannitol as cryoprotectant in the lyophilization process, different concentrations of mannitol were assessed to select the optimum concentration that facilitate the formulation of lyophilized ITZ-ZNPs with accepted PS, PDI, and ZP. Mannitol is a low molecular weight non-reducing sugar which becomes crystalline during lyophilization; such crystallinity provokes a mechanical stress that reduces the space available for the nanoparticles interactions with each other and thus prevent their aggregation [91]. Additionally, it was reported that mannitol surrounds the surface of the NPs and mask their surface charges, responsible for destabilization of the system; thus, mannitol prevents nanoparticles aggregation and increase particle stability during the lyophilization process [92]. Also, mannitol is a non-reducing sugar, thus, Maillard reactions will not occur. Maillard reactions are a chemical reactions that occur during lyophilization of protein-containing nano formulations between amino acid of the protein (zein) and the reducing sugars (cryoprotectant) [26].

Thus, characterization of the lyophilized ITZ-ZNPs was done for freshly prepared ITZ-ZNPs after addition of different mannitol concentrations in comparison to that of ITZ-ZNP before lyophilization, in triplicate, and their standard deviations were calculated and recorded in Table 4. Results demonstrated that absence of mannitol during the lyophilization process leads to the formation of unstable large aggregates of ZNPs due to mechanical stress exerted during freezing of NPs; thus, addition of mannitol before lyophilization is essential [93]. However, increasing mannitol concentration caused corresponding increase of PS and PDI and reduction in ZNPs positive charges. This may be due to crystallization of mannitol on ZNPs surface during lyophilization process that masks ITZ-ZNP-positive charges [94]. Therefore, increasing mannitol concentration led to reduction of ZP values to 17.1 ± 0.4 and 4.125 ± 0.345 at 0.25% and 0.5% mannitol, respectively. Moreover, increasing mannitol concentration to 1% converted surface charge of the lyophilized ITZ-ZNPs to become negatively charged (−4.28 ± 0.497). These results were similar to that of Zhang et al*.* in which increasing mannitol concentration cause formation of unstable large size ZNPs [93]. Also, Gagliardi et al. found that Brij O10- zein nanoparticles had a positively charged surface that close to neutrality, suggesting difficulty in formulating stable lyophilized ZNPs [26].

**Table 4:** PS, PDI, and ZP of lyophilized ITZ-ZNP optimized formulation with different mannitol concentrations compared to the same formulation before lyophilization

	Before lyophilization	After lyophilization			
		**0% Mannitol**	**0.25% Mannitol**	**0.5% Mannitol**	**1% Mannitol**
PS	208 ± 4.29	705.9 ± 141.2	240.4 ± 17.18	466 ± 50	595.9 ± 84.35
PDI	0.35 ± 0.04	0.86 ± 0.14	0.578 ± 0.02	0.697 ± 0.056	1 ± 0
ZP	35.7 ± 1.65	−0.799 ± 0.03	17.1 ± 0.4	4.125 ± 0.345	−4.28 ± 0.497

Therefore, 0.25% mannitol was preferred to preparing a stable lyophilized formulation with accepted PS and PDI values. Afterward, the lyophilized ITZ-NPs was loaded into colon-targeting capsules for further investigations.

**Fig. 9:**
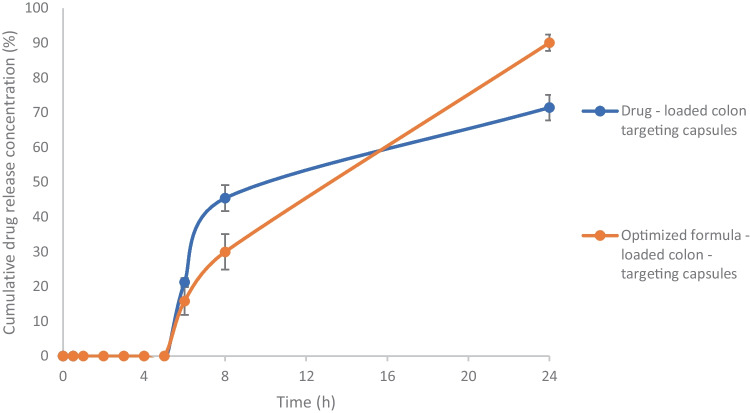
In vitro drug release of ITZ from the optimized ITZ-ZNP-loaded colon-targeted capsules compared to the release from pure ITZ-loaded colon-targeted capsules, in simulated gastric pH (2 h), simulated intestinal pH (3 h), then simulated colon pH (19 h) at 50 rpm and a temperature of 37 ± 0.5 °C. Each point represents mean ± SD (*n* = 3)

The in vitro cumulative release study of ITZ from colon-targeting coated capsules loaded with optimized ITZ-ZNP formulation was performed in comparison to that loaded with pure ITZ as represented in Fig. 9. It is obvious that drug release profile of the optimized ITZ-ZNPs-loaded colon-targeting coated capsules and that of the drug-loaded capsules showed no drug release during the first 5 h in pH 1.2 and pH 6.8 indicating that coating the loaded capsules with Eudragit^®^ S100 resulted in the formulation of successful efficient pH-dependent colon-targeting systems. Whereas once reached pH 7.4, the cumulative drug release showed abrupt release in a similar pattern to what was previously observed in ITZ release of from the optimized formulation at pH 7.4; ITZ released reached 21.3 ± 1.1% and 15.8 ± 4% after 1 h from pure ITZ-loaded capsules and ITZ-ZNP-loaded capsules, respectively, due to the swift release of ITZ adsorbed on ZNP surface. Afterwards, gradual release of ITZ from ZNPs was observed that might be due to the formation of aqueous channels through ZNPs causing it to lose its integrity and allow ITZ release gradually; such phenomena indicating the ability of zein to control ITZ release when encapsulated into the ZNPs.

Accordingly, it was predicted that the ITZ-ZNP-loaded Eudragit^®^ S100-coated capsules represent successful efficient colon-targeting system that effectively prevent ITZ release in either stomach or intestine, while initiate effectual ITZ release once the system reaches the colon leading to effective ITZ concentration that can successfully eradicate opportunistic colonic fungal infections.

#### In vivo X-ray imaging of ZNPs-loaded enteric coated capsules

**Fig. 10:**
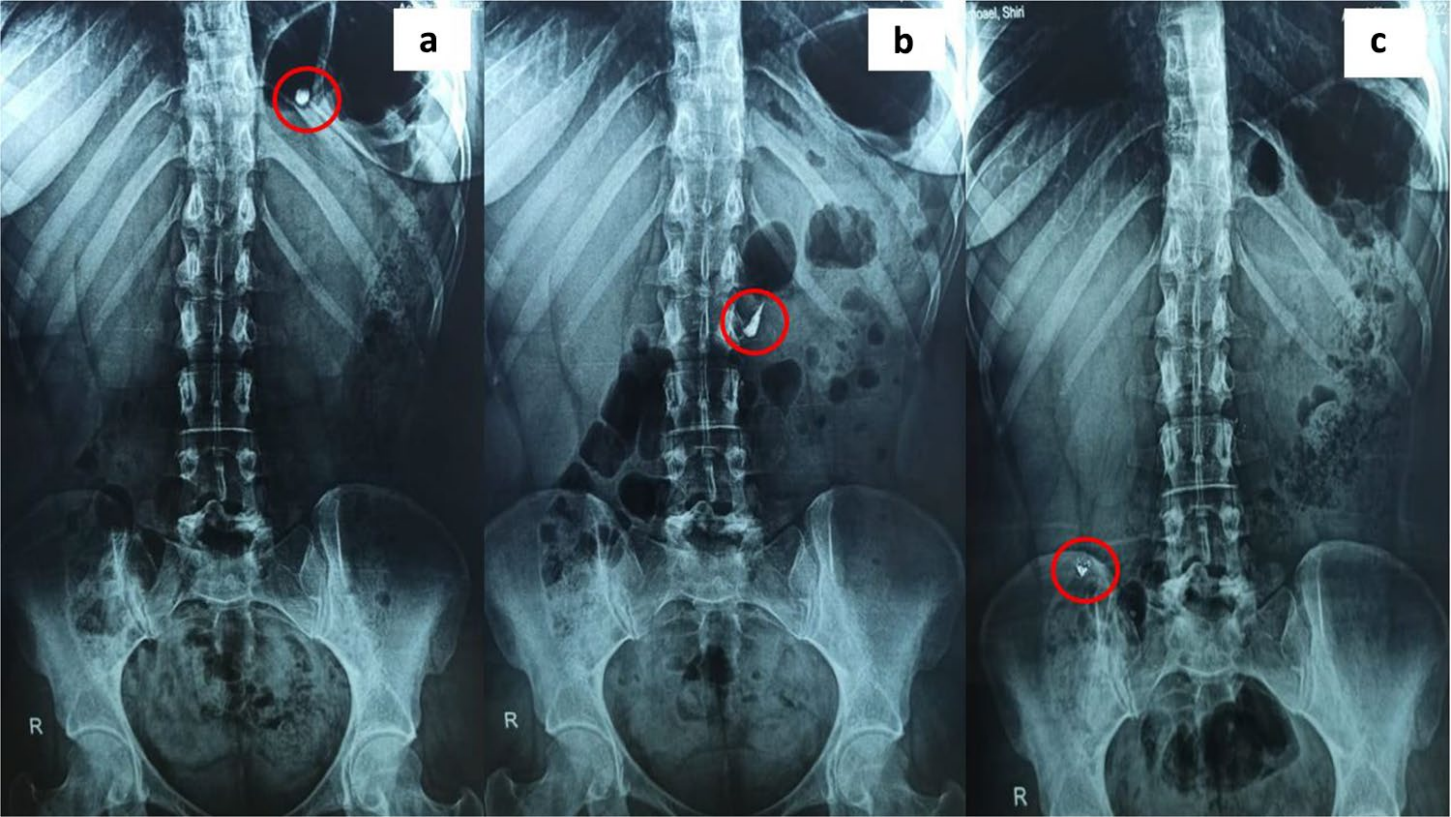
X-ray imaging of BaSO_2_-ZNPs-loaded colon-targeting capsules after oral administration in human volunteers. **a** 0.5 h post-dose, **b** 3 h post-dose, and **c** 5 h post-dose

X-ray imaging was used to track the in vivo performance of the colon-targeting capsules throughout the gastrointestinal tract. X-ray radiographs were taken after 0.5, 3, and 5 h post-oral administration of the BaSO_4_-ZNP-loaded colon-targeting capsules and presented in Fig. 10.

Following ingestion, the capsules preserved its integrity after 0.5 and 3 h post-dose as illustrated in Fig. 10a and b, respectively, which indicates the ability of the Eudragit^®^ S100-coated capsules to protect its content from gastric and intestinal fluids till reaching the colon. On the other hand, Fig. 10c shows abdominal radiographs that were taken 5 h post-dose; it is evident that the capsules start to lose their integrity and get smaller, leading to releasing of ITZ-ZNPs in the colon fluids [95]. This may be contributed to solubilization of Eudragit^®^ S100 coat when reaching the colon as it dissolves only in pH < 7 [96].

So, the Eudragit^®^ S100-coated capsules remains intact throughout the gastric and intestinal regions till reaching ileocecal region where pH become 7.4 [97], where Eudragit^®^ S100 coat dissolves and the capsules contents (ITZ-loaded ZNP) are released into the colonic media. But due to zein insolubility, it is suggested that zein start to swell and drug release gradually upon reaching the colon.

Thus, study proved that the prepared system provides promising nanoparticulate system that is able to protect ITZ throughout the GIT; preventing its release in either gastric or intestinal fluids; and thus targeting its release to the colon in order to exert effectual focused local action for the treatment of colon fungal infections.

## Conclusion

Results of the present study revealed that colon-targeted ITZ-ZNP-loaded Eudragit^®^ S100-coated capsules could represent a potential platform for the effective treatment of colon fungal infections. The current study successfully investigated the preparation of ITZ-loaded ZNPs using antisolvent precipitation technique. Central composite face-centered design (CCFD) was adopted to prepare ITZ-loaded ZNPs where different zein:drug and aqueous:organic ratios were used, followed by statistical analysis and optimization of the results using the lowest PS and PDI, highest ZP and EE% as determining criteria. Statistical optimization led to an optimized ITZ-ZNP formulation using 5.5:1 zein/drug and 9.5:1 aqueous:organic with 0.755 desirability.

Further studies illustrated that in vitro release of ITZ from optimized ITZ-ZNP formulation was enhanced in simulated colon pH than that of pure drug. TEM showed that spherical core–shell structure of the optimized ITZ-ZNP formulation with smooth surfaces and uniform size, while DSC illustrated transformation of ITZ from crystalline to amorphous state that was molecularly dispersed throughout the formulated ZNPs. Also, FT-IR analysis indicated coupling of NH of zein with the carbonyl group of ITZ through hydrogen bond without affecting ITZ antifungal activity. This was evidenced by an antifungal activity study that ensured superior antifungal activity enhancement of ITZ-ZNPs over that of aqueous ITZ suspension. Also, biosafety of the optimized formulation was confirmed by histopathological examination and SRB assay. Then, it was loaded in capsules coated with Eudragit^®^ S100 and in vitro release study suggested their ability to prevent ITZ release either in simulated stomach pH or simulated intestinal pH. Moreover, in vivo X-ray imaging showed satisfying in vivo performance of the coated capsules along the GIT as it proved their ability to protect ITZ-ZNPs throughout the GIT till reaching the colon where Eudragit^®^ S100 coat starts to dissolve releasing the capsule-loaded ITZ-ZNPs.

Accordingly, the study suggests a promising colon-targeting system, encapsulating hydrophobic drugs (ITZ), that exert effective local antifungal effect against colon fungal infections with confirmed biosafety to the colon tissue.

## Data Availability

The datasets generated during and/or analyzed during the current study are available from the corresponding author on reasonable request.
